# Correction: Molecular epidemiology and microbiological characteristics of *Cryptococcus gattii* VGII isolates from China

**DOI:** 10.1371/journal.pntd.0011100

**Published:** 2023-01-26

**Authors:** Xuelei Zang, Weixin Ke, Lifeng Wang, Hua Wu, Yemei Huang, Hengyu Deng, Meng Zhou, Ningxin Wu, Linqi Wang, Xinying Xue, Dingxia Shen

Linqi Wang is not included in the author byline. Linqi Wang should be listed as the ninth author and affiliated with State Key Laboratory of Mycology, Institute of Microbiology, Chinese Academy of Sciences, Beijing, China. The contributions of this author are as follows: Resources, Conceptualization.
